# Performance of neuroretinal rim thickness measurement by Cirrus high-definition optical coherence tomography in myopic eyes

**DOI:** 10.1038/s41598-023-39701-6

**Published:** 2023-08-05

**Authors:** Andrew K. C. Lam, H. C. Lai, Y. K. Sung, W. H. Lam, C. M. Tiu

**Affiliations:** 1 School of Optometry, The Hong Kong Polytechnic University, Kowloon, Hong Kong SAR China; 2 School of Optometry, Centre for Myopia Research, The Hong Kong Polytechnic University, Kowloon, Hong Kong SAR China; 3 Centre for Eye and Vision Research (CEVR), 17W Hong Kong Science Park, Shatin, Hong Kong SAR China; 4 Research Centre for SHARP Vision (RCSV), The Hong Kong Polytechnic University, Kowloon, Hong Kong SAR China

**Keywords:** Health care, Tomography

## Abstract

Neuroretinal rim (NRR) measurement can aid the diagnosis of glaucoma. A few studies reported that Cirrus optical coherence tomography (OCT) had NRR segmentation errors. The current study investigated segmentation success of NRR in myopic eyes using the Cirrus built-in software and to determine the number of acquisitions required to identify NRR thinning. Right eye of 87 healthy adult myopes had an optic disc scanned using Cirrus HD-OCT for five successive acquisitions. A masked examiner evaluated 36 radial line images of each scan to screen for segmentation errors using the built-in software at the Bruch’s membrane opening (BMO) and/or internal limiting membrane (ILM). Participants with three accurate NRR acquisitions had their average NRR thickness determined. This result was compared with average of the two acquisitions and the first acquisition. Among 435 OCT scans of the optic disc (87 eyes × 5 acquisitions), 129 (29.7%) scans had segmentation errors that occurred mainly at the ILM. The inferior-temporal and superior meridians had slightly more segmentation errors than other meridians, independent of axial length, amount of myopia, or presence of peripapillary atrophy. Sixty-five eyes (74.7%) had at least three accurate NRR measurements. The three acquisitions had high reliability in NRR thickness in the four quadrants (intraclass correlation coefficient > 0.990, coefficient of variation < 3.9%). NRR difference between the first acquisition and the average of three acquisitions was small (mean difference 2 ± 13 μm, 95% limits of agreement within ± 30 μm) among the four quadrants. Segmentation errors in NRR measurements appeared regardless of axial length, amount of myopia, or presence of peripapillary atrophy. Cirrus segmentation lines should be manually inspected when measuring NRR thickness.

## Introduction

Retinal nerve fiber layer (RNFL) thickness and ganglion cell analysis are key parameters used to aid the diagnosis and monitoring of glaucoma. Cirrus high-definition (HD) optical coherence tomography (OCT) (Carl Zeiss Meditec AG, Dublin, CA) is a spectral-domain OCT that provides both measures together with neuroretinal rim (NRR) thickness. NRR thickness is defined as the shortest distance between the Bruch’s membrane opening (BMO) and the internal limiting membrane (ILM). The Cirrus HD-OCT algorithm automatically detects the margins of the BMO and ILM and presents the data as the NRR thickness and area^1,2^. Hwang and Kim^3^ and Kim and Park^4^ reported that the NRR thickness from Cirrus HD-OCT had an accurate glaucoma diagnostic capability. Tai et al.^5^ suggested that the NRR thickness is a better parameter than RNFL and ganglion cell layer thickness for the detection of glaucoma.

Although disc margin can be identified from fundoscopy, BMO should be used to measure NRR thickness^6^. Unlike the measurement of NRR thickness using other OCT machines, Spectralis (Heidelberg Engineering, GmbH, Germany) for example, the BMO and ILM determined by Cirrus HD-OCT cannot be adjusted manually. Because NRR thickness measurement in Cirrus HD-OCT solely depends on the automatically identified BMO and ILM, the accuracy and consistency of detecting these two locations are important. In healthy individuals, myopic eyes may result in inconsistencies in BMO location detection, leading to inaccurate and unreliable NRR measurements^7^. Errors are more common in eyes with peripapillary atrophy (PPA)^7,8^.

Owing to the lack of manual adjustment of BMO and ILM locations, built-in NRR thickness measurement in Cirrus HD-OCT has not been well utilized clinically^3–5,7,8^. To optimize the clinical application of the NRR thickness, the repeatability of NRR measurements requires further investigation. This study aimed to investigate the frequency of errors in identifying the BMO and ILM; and factors associated with measurement errors. This study further evaluated whether taking an average from multiple NRR acquisitions would be better than taking a single measurement to determine difference in NRR thickness.

## Methods

Healthy myopes aged ≥ 18 years were recruited. The inclusion criteria were (1) clear ocular media, (2) spherical refractive error between − 0.50 D and − 10.00 D, and astigmatism not more than − 3.00 D, (3) best-corrected visual acuity of at least 0.10 logMAR, (4) intraocular pressure within 21 mmHg, (5) normal optic disc from fundoscopy (without disc haemorrhages, notches, neuroretinal rim thinning, pallor, or > 0.2 cup-to-disc ratio asymmetry between two eyes), and (6) no family history of glaucoma. Participants with ocular anomalies were excluded. The study adhered to the tenets of the Declaration of Helsinki and was approved by the Institutional Review Board of The Hong Kong Polytechnic University. Written informed consent was obtained from all participants prior to data collection.

Each participant underwent optic disc imaging using the 200 × 200 Optic Disc Cube of the Cirrus HD-OCT 5000. It has a scan speed of 27,000 A-scan/B-scan. Motion tracking and fixation were controlled using a built-in FastTrac™ retinal tracking system. Scans were repeated until the minimum signal strength reached 7. Five successive scans were acquired without using a mydriatic agent. A one-minute break was provided between successive scans. The NRR thickness profiles were saved and exported for further analysis.

### Statistical analysis

Measurements were taken in both eyes, and only data from the right eye were analysed. An experienced examiner blinded to the participants’ details evaluated the quality of each Optic Disc Cube acquisition, which included 36 radial scans centred at the disc. The evaluation focused on segmentation errors, if any, in the NRR. This included the BMO and ILM identification. When the BMO and/or the ILM marked by the built-in software were not in their proper locations in the colour code OCT images, segmentation error was declared. The frequency of segmentation errors from five successive scans was determined, and factors that might be associated with segmentation errors were identified. Eight meridians were used to classify the locations of segmentation errors: superior, superior nasal, nasal, inferior nasal, inferior, inferior temporal, temporal, and superior temporal. One sample proportion test was applied to determine whether segmentation errors appeared to be more frequent in certain meridians than others.

Eyes were categorized into two groups, having segment errors or being error-free in OCT acquisitions. The two groups were compared in terms of age, gender distribution, spherical equivalent refraction, axial length, and the presence of PPA. For continuous quantitative variables (age, spherical equivalent refraction, and axial length), the Mann–Whitney U test or unpaired t-test was used after checking normality of the data using the Kolmogorov–Smirnov test. Chi-square was used for categorical variables (age and presence of PPA).

The average from three Optic Disc Cube acquisitions without errors was assumed to provide the most accurate estimate of NRR thickness. The NRR thickness at the BMO was divided into 180 measurement points 2° apart. In addition to the overall NRR thickness around the optic disc, 180 measurement points were grouped as temporal (0–22 points), superior (23–67), nasal (69–112), inferior (113–157) and back to temporal (158–179). Differences in NRR thickness among the three acquisitions were studied using intraclass correlation coefficients with a two-way mixed model (ICC). The within-subject standard deviation (Sw) was calculated. The repeatability coefficient of the NRR from the three measurements was 2.77 × Sw. The coefficient of variation (CoV) of the NRR thickness, which is the ratio of the standard deviation to the mean, from three measurements was also calculated. Agreement was compared between average of three OCT acquisitions and the first acquisition, and compared with average of two OCT measurements using the 95% limits of agreement (LoA). Confidence intervals for the LoA were also calculated^9^. All statistical analyses were performed using the Statistical Package for the SPSS version 26.0 (IBM Corp., USA).

## Results

The optic discs of 87 participants were scanned (Table 1). Only the right eye was analysed because the refractive error and axial length of the two eyes were highly correlated.

**Table 1:** Demographic information of 87 participants.

Age	21.0 (2.0) years	
Gender	43 male/44 female	
SER (D): right eye	− 4.50 (2.50)	Spearman’s rho = 0.856, p < 0.001
Left eye	− 4.62 ± 2.28	
AL (mm): right eye	25.46 ± 1.11	Pearson correlation = 0.906, p < 0.001
Left eye	25.44 ± 1.10	

Results are presented as mean ± standard deviation or median (interquartile range).

*SER* spherical equivalent refraction, *AL* axial length.

**Figure 1:**
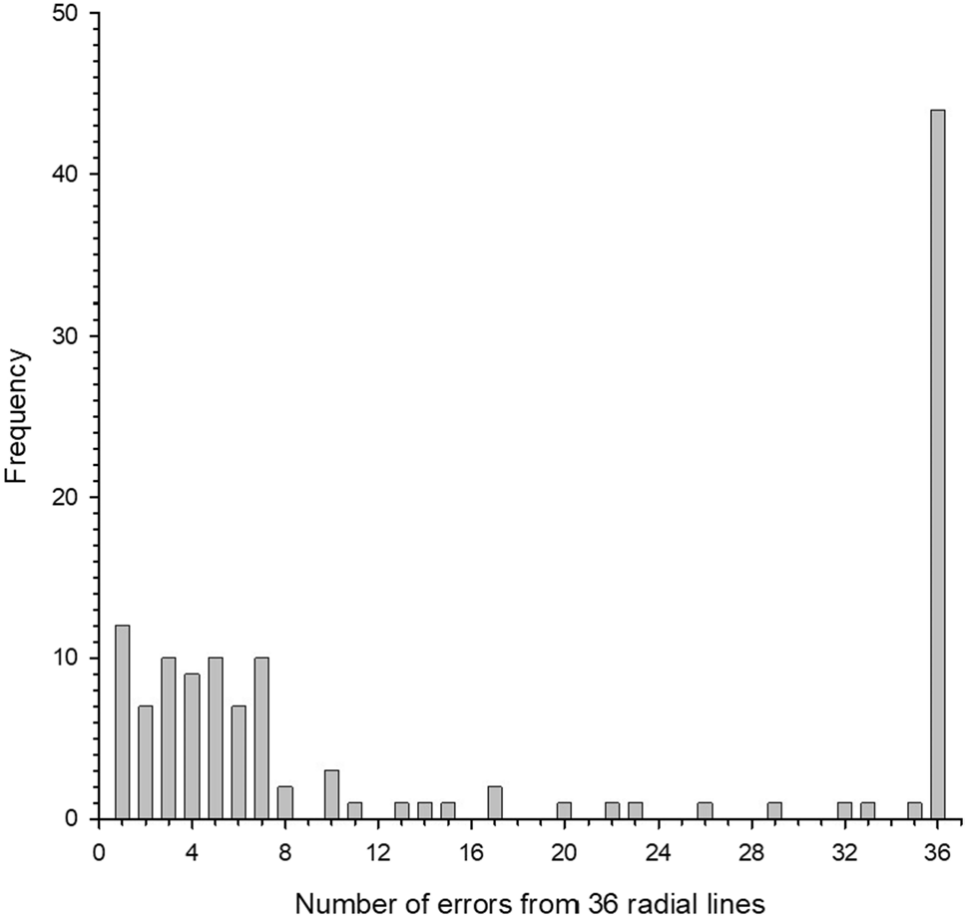
Frequency of having segmentation errors in each OCT scan.

There were 435 OCT scans (87 eyes × five acquisitions) and 15,660 OCT line images (36 radial lines per scan). Segmentation errors occurred in 2191 line images (14.0%) when generating the NRR, which involved 129 scans (29.7%). Figure 1 shows a frequency plot about segmentation errors for 36 radial lines.

**Figure 2:**
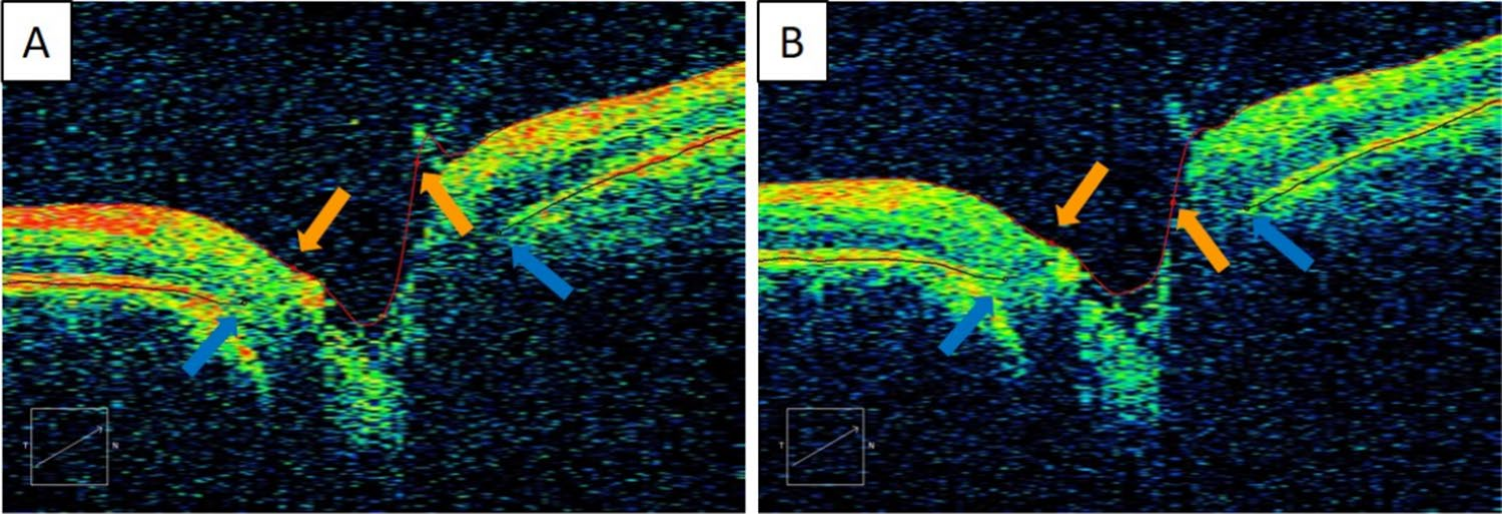
Representative case showing segmentation error. (**A**) Segmentation error in ILM along the 30° meridian in the 1st acquisition. (**B**) The ILM error did not happen in the 2nd acquisition. BMO is indicated by the blue arrow, ILM is indicated by the orange arrow. Neuroretinal thickness is measured between the black and red dots.

In 44 scans, all 36 radial lines exhibited segmentation errors. Almost all errors appeared at the ILM (125 scans), one scan had errors at the BMO, and three scans had errors in both the ILM and BMO. Figure 2 shows an example of segmentation error in the ILM of one eye.

The frequency of segmentation errors in eight meridians is shown in Table 2. Only the superior meridian had marginally more segmentation errors than the other meridians (one sample proportion test, p = 0.049).

**Table 2:** Frequency of segmentation errors at different meridians.

Meridian	Scan count	Percentage
Superior	69	15.6
Superior nasal	53	12.0
Nasal	42	9.5
Inferior nasal	51	11.5
Inferior	61	13.8
Inferior temporal	65	14.7
Temporal	50	11.3
Superior temporal	51	11.5

Total count = 442 (> 435) because a few eyes had errors in more than one meridian.

Forty-two eyes (48.3%) showed no errors in all five acquisitions. Among 45 eyes with segmentation errors, 17 eyes had errors in one acquisition, six eyes had errors in two acquisitions, three eyes had errors in three acquisitions, three eyes had errors in four acquisitions, and 16 eyes could not obtain an error-free scan in all five acquisitions. Comparing 42 eyes with error-free NRR segmentation to 45 eyes with segmentation errors, the two groups had similar age (median 22 vs 21 years, Mann–Whitney U test, p = 0.351), gender distribution (male/female = 20/22 vs 23/22, Chi-square, p = 0.745), presence of PPA (presence/absence = 31/11 vs 33/12, Chi-square, p = 0.960), spherical equivalent refraction (mean − 4.57D vs − 4.74D, unpaired t-test, p = 0.716), and axial length (mean 25.50 mm vs 25.42 mm, unpaired t-test, p = 0.728).

Sixty-five eyes (74.7%) had at least three error-free optic disc cube acquisitions. Reliability of the first three error-free optic disc cube acquisitions is shown in Table 3. It exhibited excellent reliability with an ICC of > 0.99. The repeatability coefficient varied from 27.7 to 44.5 μm in different quadrants. CoV was less than 4%.

**Table 3:** Reliability of neuroretinal rim thickness from three error-free optic disc cube scans.

	Intraclass correlation coefficient	Repeatability coefficient (μm)	Coefficient of variation (%)
Overall	0.997	22.4	2.07
Inferior	0.992	44.5	3.64
Superior	0.993	44.2	3.86
Nasal	0.995	39.1	3.32
Temporal	0.993	27.7	3.44

**Figure 3:**
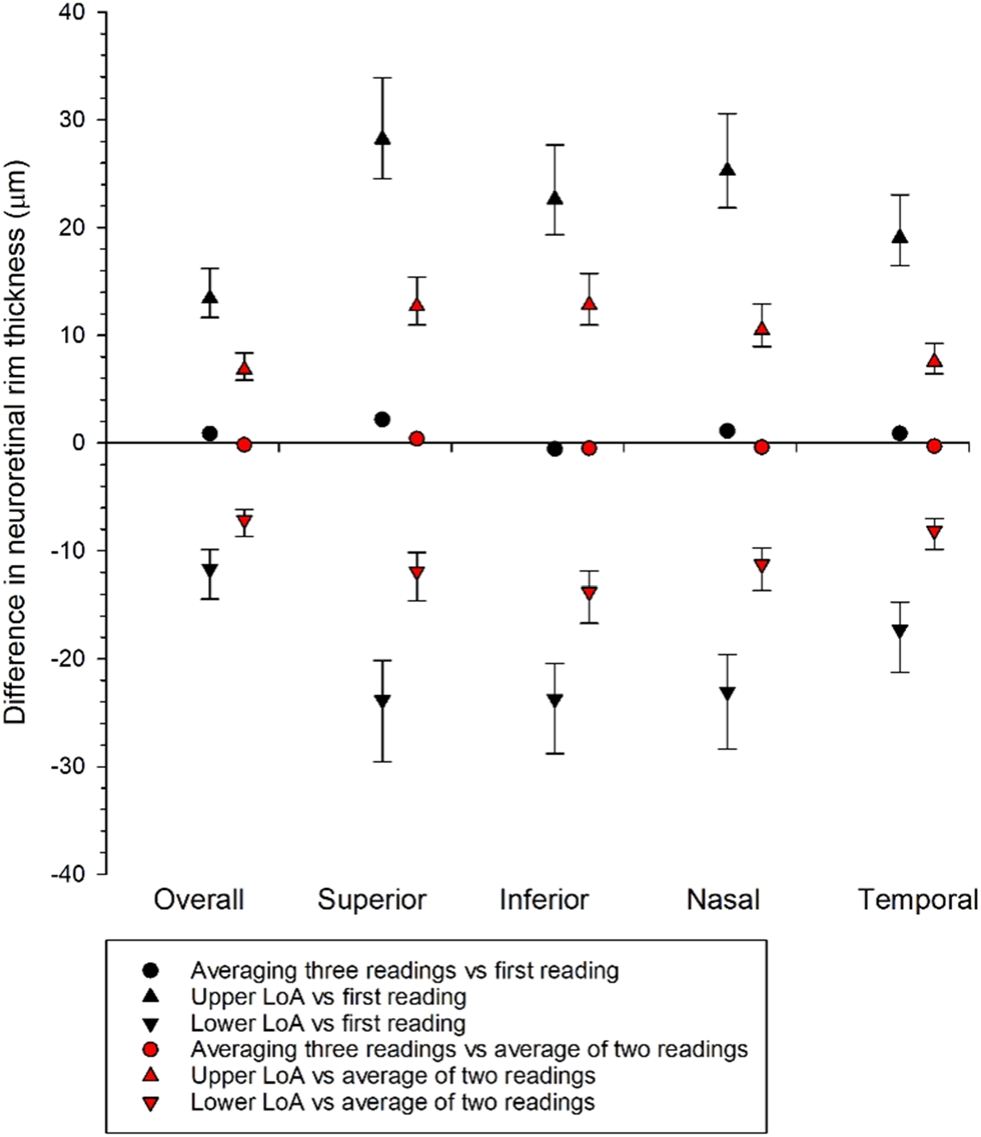
Mean difference and agreement of neuroretinal rim (NRR) thickness using different numbers of NRR acquisitions at the four quadrants and the Overall NRR. The upper limits of agreement (LoA) when compared with the first reading for the Overall, Superior, Inferior, Nasal, and Temporal quadrants were 13.4 μm, 28.2 μm, 22.6 μm, 25.3 μm, and 19.1 μm, respectively; the lower LoA were − 11.7 μm, − 23.8 μm, − 23.7 μm, − 23.1 μm, and − 17.3 μm, respectively. The upper LoA when compared with average of two readings for the Overall, Superior, Inferior, Nasal, and Temporal quadrants were 6.8 μm, 12.7 μm, 12.8 μm, 10.5 μm, and 7.6 μm, respectively; the lower LoA were − 7.1 μm, − 11.9 μm, − 13.8 μm, − 11.3 μm, and − 8.2 μm, respectively. Error bars represent the exact 95% confidence intervals for the upper and lower LoA.

Figure 3 shows mean difference and agreement of NRR comparing different averaging conditions of the overall NRR thickness and at the four quadrants. Averaging two acquisitions provided better agreement to the average of three acquisitions than using the first acquisition, which is conceivable. Agreements using just the first acquisition were within ± 30 μm at different quadrants.

Overall NRR thickness and that at the four quadrants of 65 eyes using average from three acquisitions are shown in Table 4. There were only 14 eyes (22%) following the inferior ≥ superior ≥ nasal ≥ temporal (ISNT) rule but 46 eyes (71%) following the inferior > superior > temporal (IST) rule.

**Table 4:** Neuroretinal rim (NRR) thickness, mean ± standard deviation, from average of three error-free optic disc cube scans.

	NRR thickness (μm)		
Overall	391.5 ± 90.9	Follow ISNT	Follow IST
Inferior	441.5 ± 104.6	14 eyes (22%)	46 eyes (71%)
Superior	412.5 ± 110.6		
Nasal	424.7 ± 120.7		
Temporal	290.4 ± 70.9		

*ISNT* inferior ≥ superior ≥ nasal ≥ temporal. *IST* inferior > superior > temporal.

## Discussion

Neuroretinal rim thickness may provide better information than RNFL to aid the diagnosis of glaucoma^3^. Kim and Park^4^ found that NRR could reduce false-positive results indicated by RNFL. NRR also has good diagnostic utility in pre-perimetric glaucoma^5^. Accurate NRR thickness measurement depends on the correct identification of the BMO and ILM. The current study found a high number (almost 30%) of segmentation errors from the Cirrus algorithm which mainly affected the ILM identification. The reasons for this was the presence of vitreous floaters or glial tissues at the optic disc.

Hwang et al.^10^ reported that disc detection errors mainly occur in the superior and temporal quadrants. Predominately, segmentation errors appear in highly myopic eyes with PPA^7,10^. Most of our participants had PPA, but the presence of segmentation errors was not associated with the presence of PPA. We also found no significant effect of gender, amount of myopia, and axial length on segmentation errors. Hwang et al.^7^ further reported that the Cirrus algorithm misidentified the scleral bed as the BMO, especially when the scleral bed had sloping or stepping contour changes. We are also concerned when more errors appear in the superior and inferior-temporal regions, which are the primary sites of glaucomatous neuroretinal rim damage^11^.

Although RNFL thickness is commonly used for glaucoma diagnosis and monitoring, neuroretinal rim thickness may be more useful in certain circumstances. For example, the epiretinal membrane at the peripapillary region could affect RNFL measurement^12^. Eyes with myopia^13^ or tilted disc^14^ demonstrate more false-positive results when using RNFL than when using minimum neuroretinal rim thickness. In early normal tension glaucoma, rate of neuroretinal rim thinning was also faster than RNFL did^15^. Structure–function relationship was stronger with minimum neuroretinal rim thickness than RNFL thickness^16^. Most OCT machines provided neuroretinal rim area as well as thickness. The rim area could be better than the rim thickness in eyes with different optic disc sizes because of the influence of disc size on rim thickness^17^.

Spectralis OCT (Heidelberg Engineering, GmbH, Germany) is another machine that provides neuroretinal rim thickness. It represents the minimum rim thickness; hence, it is called Bruch’s membrane opening-minimum rim width (BMO-MRW). The neuroretinal rim thickness generated by the Cirrus algorithm may not represent the thinnest rim measured from the BMO^16^. Among 129 scans with segmentation errors, 126 scans exhibited neuroretinal rim thickness exceeding the 5th percentile of the built-in normative database (with a green code). Three scans had rim thickness with a yellow code (borderline or < 5% deviation from the normative database). Since the preponderance of segmentation errors occurred at the ILM, rim thickness could be over-estimated, leading to false negatives. Due to artefacts in OCT images, previous studies found that OCT metrics could be erroneously interpreted as abnormally thin, i.e. false positive or called ‘red disease’^18^. Kim and Park^4^ found that red disease was more prevalent when RNFL was used, whereas neuroretinal rim thickness provided a more accurate diagnosis of glaucoma. One drawback of the Cirrus algorithm is that the two landmarks (BMO and ILM) cannot be manually adjusted, as the Spectralis OCT allows. It is difficult to corroborate whether the rim thickness found in the current study was within the normative database. To evaluate the likelihood of segmentation errors in glaucoma patients when measuring neuroretinal rim thickness, future research should include glaucoma patients. Due to segmentation error at the ILM, false negatives or ‘green disease’ pose the greatest concern^19^. The current study excluded 22 eyes because fewer than three acquisitions were free of segmentation errors, leaving 65 eyes for analyses. Although manual adjustment of the BMO is feasible in the Spectralis OCT, Zheng et al.^20^ found that highly myopic eyes commonly have indiscernible BMO at the temporal, superior-temporal, and inferior-temporal meridians. This may compromise the diagnostic value of neuroretinal rim thickness.

The reliability of the three successive NRR measurements was excellent, with an ICC above 0.99. The intrasession repeatability was very good, with a CoV of < 4% (Table 3). Are three measurements required to calculate an average, or are two measurements, or even a single measurement, adequate? This depends on the purpose of the NRR measurement. Hwang and Kim compared the NRR of glaucoma and healthy participants using the Cirrus HD-OCT^3,21^. The smallest difference in NRR between mild glaucoma and control was at the ninth clock-hour or temporal sector (73 μm) and the greatest at the 6th clock-hour or inferior sector (177 μm)^3^. When NRR was grouped into four quadrants, the difference in NRR was the smallest at the temporal quadrant (81 μm) and the greatest at the inferior quadrant (159 μm)^21^. Moderate to advanced glaucoma had an even thinner NRR. Kim and Park^4^ reported similar findings when comparing myopic glaucoma patients with healthy participants. The NRR in the temporal quadrant had a difference of 70 μm and 157 μm in the inferior quadrant. Tai et al.^5^ compared the NRR of pre-perimetric glaucoma patients with that of healthy participants. The temporal or ninth clock-hour sector had the smallest difference of 50 μm, whereas the difference at the sixth clock-hour or the inferior sector was 111 μm. They also identified the best diagnostic value in the NRR thickness in the inferior quadrant. Figure 2 shows an agreement of within ± 15 μm between the average of two NRR measurements and the reference standard (average of three error-free NRR measurements with a signal strength of at least 7). It was shown that a single NRR measurement could still provide acceptable agreement (within ± 30 μm) when compared to the standard. Therefore, a single NRR measurement may be sensitive enough to detect NRR thinning if NRR is used for glaucoma evaluation. Dependent on the application of NRR, various averaging methods may be used.

This study also compared the NRR thicknesses in the four quadrants. Only 22% of eyes had inferior ≥ superior ≥ nasal ≥ temporal rim thickness (ISNT rule). However, over 70% of the eyes followed the inferior > superior > temporal rim (IST pattern) after excluding the nasal quadrant. This is similar to the study by Hwang and Kim^21^ who also used Cirrus HD-OCT. Poon et al.^22^ evaluated rim thickness using colour fundus images. Only 37% of the eyes followed the ISNT rule, but 71% followed the IST pattern. The unusual thickening of the NRR could have been due to the inclusion of vascular trunks embedded within the rim tissue. Park et al.^23^ used Spectralis OCT and found that 32.5% of the subjects followed the ISNT rule using BMO-MRW. This highlights the importance of manually correcting ILM and BMO. Qiu et al.^24^ used confocal scanning laser ophthalmoscopy to measure rim area. They found moderate-to-high myopic eyes following the ISNT (62% to 64%) or IST (65% to 66%) patterns.

Morgan et al.^25^ used stereoscopic optic disc photographs and concluded that the ISNT rule had limited utility in the diagnosis of open-angle glaucoma. Since few white glaucoma patients were examined, they admitted that their results might not have been applicable to other ethnic groups. Recently, Iwase et al.^26^ completed a population-based study on Japanese patients using stereoscopic photographs of the optic disc. They found that less than 7% of normal eyes followed the ISNT rule, whereas 70% exhibited the IST pattern. The IST pattern also showed the highest positive likelihood ratio for glaucoma detection.

There were several limitations to the current study. An experienced examiner evaluated the colour code OCT images to subjectively determine segmentation errors. It could not be determined quantitatively, such as a deviation greater than a certain amount of microns from the presumed accurate location. The participants had a narrow age range (18 to 30 years). However, if vitreous floaters are the main cause of segmentation errors, young axial myopes are more likely to have vitreous floaters^27^. This study was limited by the use of a single OCT machine. Ideally, the same participants should have undergone NRR thickness measurements using different OCT machines with manual correction of the BMO and ILM. Moreover, all the participants were healthy individuals. Future studies should include myopic glaucoma patients. This study presumed that the most accurate estimation of NRR thickness would result from averaging three Optic Disc Cube acquisitions that were free of errors. Although an average result from multiple acquisitions should be more accurate, it may not be clinically practicable due to time constraints or patient fatigue. Several studies indicate that aggregating three measurements can provide a reliable reference for measurements such as perfusion density and vessel density^28^. The average of three measurements appears adequate to detect the smallest difference in pulsatile ocular blood flow between glaucoma and healthy patients^29^.

In conclusion, segmentation errors in measuring NRR can occur regardless of axial length, amount of myopia, or presence of peripapillary atrophy when Cirrus HD-OCT is used. To obtain an accurate NRR measurement, one good acquisition might be enough provided that if the acquisition is free from segmentation error.

## Data Availability

The datasets used and/or analysed during the current study available from the corresponding author on reasonable request.
